# Spontaneous activation of cortical somatosensory networks depresses their excitability in preterm human neonates

**DOI:** 10.1162/IMAG.a.1324

**Published:** 2026-08-06

**Authors:** Kimberley Whitehead, Maria Pureza Laudiano-Dray, Judith Meek, Sofia Olhede, Lorenzo Fabrizi

**Affiliations:** Research Division of Digital Health and Applied Technology Assessment (DHATA), King’s College London, London, United Kingdom; Department of Neuroscience, Physiology and Pharmacology, University College London, London, United Kingdom; Elizabeth Garrett Anderson Obstetric Wing, University College London Hospitals, London, United Kingdom; Department of Mathematics, Ecole Polytechnique Federale de Lausanne, Lausanne, Switzerland

**Keywords:** delta brushes, spontaneous activity transients, brain development, neuronal refractory period, somatosensory evoked potentials, early gamma oscillations

## Abstract

The cortical activity of preterm human infants is highly discontinuous, comprising transient high-amplitude bursts separated by periods of relative quiescence. While the functional significance of these bursts is well established, the underlying mechanism remains unclear. This burst–quiescence pattern could arise from a transient refractoriness within excitatory recurrent cortical networks following spontaneous activation. To assess this possibility, we tested whether such activation is followed by a transient reduction in excitability by evaluating whether externally evoked tactile responses are attenuated when somatosensory circuits have recently been active. We recorded electroencephalographic (EEG) responses to tactile stimulation of hands and feet in 35 preterm infants (40% female), with a median postmenstrual age of 32 weeks. This stimulation elicited wideband increases in EEG power, showing two distinct peaks: one in the delta range (1 Hz) and another in the alpha-beta range (~13 Hz). Low-frequency activity showed a single, broadly distributed peak across the scalp, whereas faster high beta–gamma responses were more confined to somatotopically specific regions, suggesting engagement of both widespread (tangential) and localized (columnar) cortical circuits. Importantly, the magnitude of the evoked response was significantly reduced when the activity immediately preceding stimulation resembled the spectro-spatial pattern of the somatosensory evoked response, indicating prior spontaneous activation of the somatosensory network. Stimulus-evoked EEG power changes decreased by 3.2 and 2.5 dB following hand and foot stimulation, respectively, for every 1.0-degree increase in the similarity between pre-stimulus activity and the spectro-spatial pattern of the somatosensory evoked response (scale 0–5). This effect was the strongest and most temporally sustained at slower frequencies. These results suggest that when somatosensory networks are spontaneously active, they become temporarily less responsive to stimulation—a form of refractoriness—preventing immediate reactivation. The extent of this refractory-like modulation is not uniform but depends on the spatial scale of the underlying networks, as indexed by their dominant frequency of activation. This mechanism may explain the cyclical pattern of bursting and quiescence neural activity observed in the preterm brain.

## 1 Introduction

Organized spontaneous activity across the central nervous system is a characteristic feature of developing vertebrate neuronal circuits. This activity is necessary for initial development of neuronal ensembles and, in the cortex, engages columnar and extended networks, establishing the template of the mature functional architecture before the arrival of sensory signals (Martini et al., 2021; Molnár et al., 2020). This spontaneous activity is characteristically discontinuous where sudden high-amplitude bursts of synchronous neuronal activity interrupt periods of quiescent background (Khazipov & Luhmann, 2006). This cyclical pattern can be explained by an excitatory recurrent network that encounters activity-dependent depression (Dutta et al., 2023; Kirmse & Zhang, 2022; Tabak et al., 2000). Indeed, spontaneous activity transiently depresses synaptic responsiveness to incoming stimuli in embryonic spinal cord preparations (refractoriness), possibly because of transmitter depletion and ligand-induced changes in postsynaptic currents, which slowly recovers in the inter-burst interval (Fedirchuk et al., 1999). This refractory period plays a key role in defining the propagation domains of spontaneous retinal waves (Feller et al., 1996; Godfrey & Swindale, 2007) and is related to after-hyperpolarizations of amacrine cells (Zheng et al., 2006) which also occur in spinal neurons (O’Donovan, 1999). In regards to its function, the episodic nature of activity bouts is thought to provide a high signal-to-noise ratio to the circuit, which could enhance synaptic efficacy in developing networks (O’Donovan, 1999; Tiriac et al., 2014).

Early cortical activity in humans is also highly discontinuous. The preterm electroencephalograph (EEG) is characterized by high voltage transient events with rich spectro-spatial structures (delta brushes and temporal theta) separated by low-amplitude inter-burst intervals until about 35 weeks postmenstrual age (Bourel-Ponchel et al., 2021; Wallois et al., 2021). These spontaneous activity bursts occur with varying topographical configurations (Arichi et al., 2017) which sometimes resemble those evoked by external stimulation (Supplementary Fig. S1). The diagnostic and prognostic importance of this characteristic pattern of activity is well established (Guérit et al., 2009; Koskela et al., 2023; Watanabe et al., 1999; Whitehead et al., 2016); however, its aetiology, that is, why the preterm EEG is discontinuous, is not clear. This characteristic discontinuous pattern could arise, as in other species, from alternating periods of high activity followed by periods of reduced networks excitability or refractoriness. To test this, we examined whether externally evoked tactile responses are attenuated when somatosensory circuits are already engaged, and whether any such modulation reflects a refractory-like process with limited temporal duration. Because both spontaneous and stimulus-evoked activity occur across a range of oscillatory frequencies associated with different periods of network engagement (e.g. ~1 s for 1 Hz delta activity versus ~0.1 s for 10 Hz alpha activity), we hypothesized that slower oscillations may be associated with longer refractory windows. We, therefore, characterized the spectral profile of this modulation, assessing whether it varies with oscillatory frequency.

## 2 Materials and Methods

### 2.1 Subjects

Thirty-five preterm infants were recruited for this study from the neonatal wards at the Elizabeth Garrett Anderson wing of University College London Hospitals (Table 1). Ethical approval was obtained from the NHS Research Ethics Committee, and informed written parental consent was obtained prior to each study. No neonates were acutely unwell at the time of study, required mechanical ventilation, or were receiving analgesics or anti-seizure medication. Exclusion criteria included a diagnosed chromosomal abnormality, high-grade germinal matrix-intraventricular haemorrhage (grade III or above), or birth weight below the 2^nd^ centile. All EEGs were assessed as normal for postmenstrual age by a clinical neurophysiologist (KW) (Tsuchida et al., 2013).

**Table 1.** Demographics of the sample.

	Total
No. of neonates	35
No. of stimulation trains	101
Sleep state at onset of stimulation train	68 active sleep; 33 quiet sleep
Details of stimulation trains	48 hands (24 right, 24 left); 53 feet (29 right, 24 left)
No. of taps	1504
Details of taps	700 hands (350 right, 350 left), 804 feet (right 432, left 372)
Median (range) PMA at time of study (weeks+days)	32 + 6 (28 + 2–35 + 5)
Median (range) PNA at study (days)	7 (2–33)
Sex (% female)	40%
Multiple gestation neonates	31%
Median (range) birth weight (g)	1575 (785–2440)

PNA indicates postnatal age; PMA indicates postmenstrual age: gestational age at birth plus PNA; Preterm = < 37 weeks gestational age at birth.

### 2.2 EEG recording set-up

Up to 19 recording electrodes (disposable Ag/AgCl cup) were positioned according to the modified international 10/10 placement system at F3, F4, FCz, Cz, CPz, C3, C4, CP3, CP4, F7, F8, T7, T8, P7, P8, TP9, TP10, O1, and O2. A reduced number of electrodes were applied if the infant became unsettled during set-up (median 18; range 11–19). The reference electrode was placed at Fz, and the ground electrode was placed at FC5/6. Target impedance of electrodes was <10 kΩ (André et al., 2010). EEG was recorded with a direct current (DC)-coupled amplifier from DC-800 Hz using a Neuroscan (Scan 4.3) SynAmps2 EEG/EP recording system. Signals were digitized with a sampling rate of 2 kHz and a resolution of 24 bit.

### 2.3 Sensory stimulation

Tactile stimuli were delivered by tapping the infants’ hands and feet while naturally asleep in the cot or held by their parents, with a hand-held tendon hammer (Supplementary Video S1) (Worley et al., 2012). Stimulus timings were recorded using an impedance head with a built-in calibrated force transducer (Bruel & Kjaer, Type 8001, Denmark), placed between the handle and the rubber tip (Worley et al., 2012). To do this, the output of the force transducer was processed by a discriminator circuit (Fig. 2 in Worley et al. (2012)). In case a stimulation event was not detected by the hardware, it was identified by thresholding the force signal which is recorded alongside the EEG. For an illustrative example of the force signal and its deflection in correspondence of a tap, see Supplementary Figure S2, which is a still from Supplementary Video 1 in Whitehead et al. (2020).

A train of somatosensory stimuli was delivered to each limb. The interstimulus interval was large, variable, and self-paced by the experimenter (usually 8–20 s). In case the infant moved, the tap was delayed by several seconds to avoid potential modulation of the somatosensory response by the movement (Saby et al., 2016) and allow movement artefacts to resolve. The sequence in which the limbs were stimulated varied across subjects (Supplementary Table S1; Pearson’s Chi-squared test with simulated p-value (2000 replicates): χ^2^ = 4.88, p = 0.8486). We attempted to stimulate every limb at every test occasion, but this was not always possible, for example when a cannula was present, or if the infant became unsettled. This resulted in a total of 101 stimulation trains (i.e., stimulated limbs) of 2–48 taps (mean 15).

### 2.4 EEG pre-processing

The infant’s sleep state was scored in 30 s epochs using EEG, respiratory movement, and behavioral features, by a Clinical Scientist in Neurophysiology (KW) according to published criteria (Grigg-Damberger, 2016; Tsuchida et al., 2013) (see Fig. 1 in Georgoulas et al., 2021 for illustrative examples in a very preterm infant). Data pre-processing was then carried out using EEGLAB v.13 (Swartz Center for Computational Neuroscience) and custom written MATLAB functions. Bad channels (poor contact with the scalp) were removed following visual inspection from 13 datasets (Supplementary Fig. S3; median: 1 channel, range: 1–3 channels). After this, missing or bad channels were estimated with spherical interpolation as implemented in EEGLAB. EEG recordings following stimulation of the left hemi-body were ‘side-swapped’ so that recordings contralateral to the stimulation site appear on the ‘same side’ of the scalp. There are no differences between somatosensory responses to right vs. left body stimulation in preterm infants (Leikos et al., 2020).

### 2.5 Analysis of stimulus-evoked EEG power spectral density (PSD) changes

We first defined the functional engagement of somatosensory cortical networks as the spectro-spatial characteristics of the EEG power spectral density (PSD) changes elicited by tactile stimulation of hands or feet across the whole scalp. To do that, we compared the median (across all subjects i and trials j) PSD (0.2–40 Hz) of the 3 s post-stimulus segment ${\hat{S}}_{S,ij}^{\left(k\right)}\left(\omega \right)$ with that of the 3 to 0 s before stimulus onset (baseline) ${\hat{S}}_{B,ij}^{\left(k\right)}\left(\omega \right)$ at every channel k. ω denotes frequency. PSD for each trial was estimated using a periodogram approach. Each segment was tapered with the first discrete prolate spheroidal sequence before calculating the individual power periodogram. We then took the median power periodogram across all trials for the baseline and post-stimulus segments as:

$$\begin{array}{c} {\bar{S}}_{B}^{\left(k\right)}\left(\omega \right)=median_{1\leq l\leq N}\left({\hat{S}}_{B,l}^{\left(k\right)}\left(\omega \right)\right), \end{array}$$ (1)

and

$${\bar{S}}_{S}^{\left(k\right)}\left(\omega \right)=median_{1\leq l\leq N}\left({\hat{S}}_{S,l}^{\left(k\right)}\left(\omega \right)\right),$$ (2)

Here, N is the total number of trials across all subjects and ${\hat{S}}_{B,l}^{\left(k\right)}\left(\omega \right)$ and ${\hat{S}}_{S,l}^{\left(k\right)}\left(\omega \right)$ denote the same quantities as ${\hat{S}}_{B,ij}^{\left(k\right)}\left(\omega \right)$ and ${\hat{S}}_{S,ij}^{\left(k\right)}\left(\omega \right)$, respectively, after concatenating the subject index i and trial index j into a single index l.

To assess whether there was a significant difference between these quantities (i.e. whether there was a significant change in the median power periodogram following stimulation), we computed the ratio:

$${\bar{T}}^{\left(k\right)}\left(\omega \right)={\bar{S}}_{S}^{\left(k\right)}\left(\omega \right)/{\bar{S}}_{B}^{\left(k\right)}\left(\omega \right)$$ (3)

**Fig. 1.**
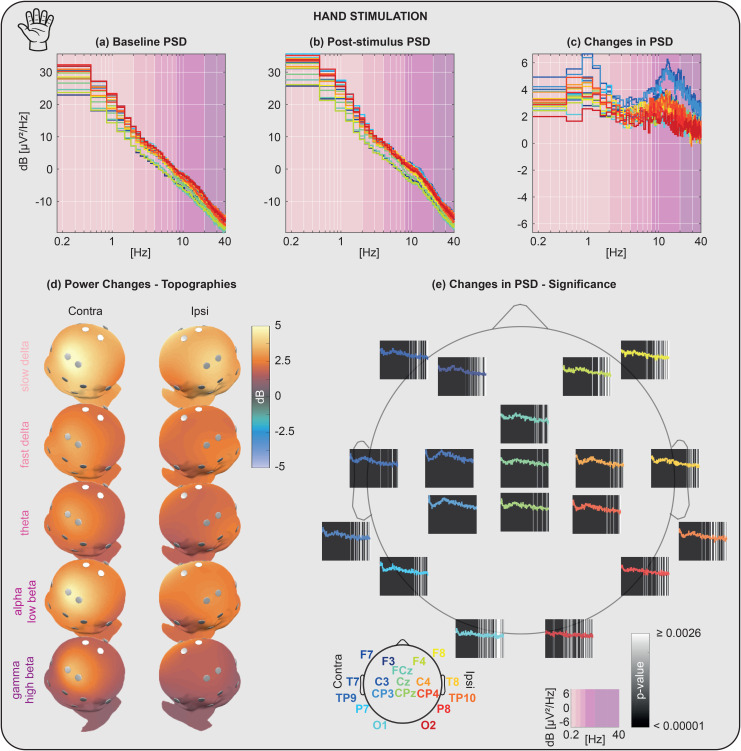
Spectro-spatial EEG power changes associated with tactile stimulation of the hands. Median power spectral density (PSD) at baseline (a) and following stimulation (b) at each channel. Changes in PSD elicited by the stimulation (c) and 3D maps of scalp distribution of power changes in the slow delta (0.2–2 Hz), fast delta (2–4 Hz), theta (4–8 Hz), alpha-low beta (8–20 Hz), and high beta-gamma (20–40 Hz) band (d). Statistical significance of the changes in PSD at each channel (e), note that the changes are the same as those in panel (c) but on a linear frequency axis to allow the display of higher frequencies. p-value limits relate to adjustment for multiple comparisons.

**Fig. 2.**
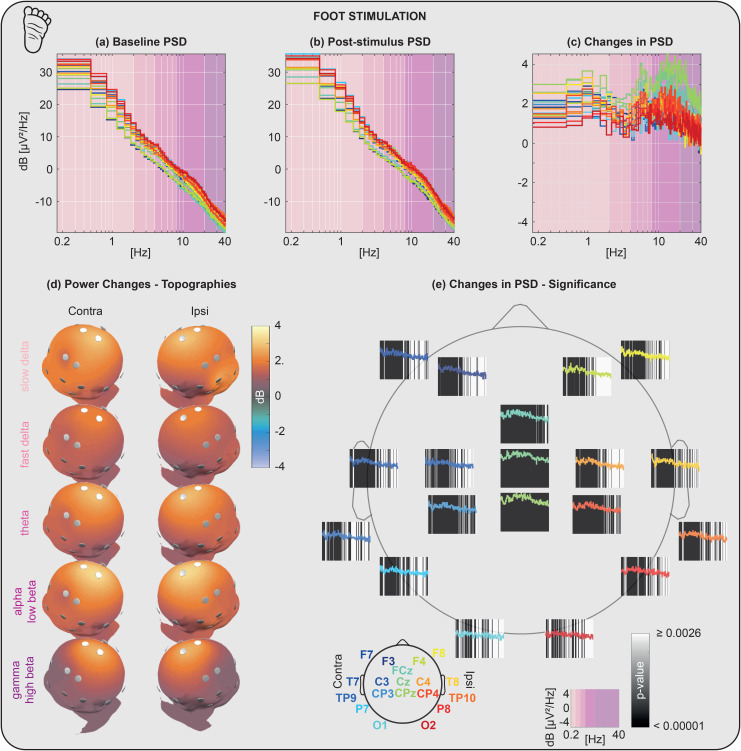
Spectro-spatial EEG power changes associated with tactile stimulation of the feet. Median power spectral density (PSD) at baseline (a) and following stimulation (b) at each channel. Changes in PSD elicited by the stimulation (c) and 3D maps of scalp distribution of power changes in the slow delta (0.2–2 Hz), fast delta (2–4 Hz), theta (4–8 Hz), alpha-low beta (8–20 Hz) and high beta-gamma (20–40 Hz) band (d). Statistical significance of the changes in PSD at each channel (e), note that the changes are the same as those in panel (c) but on a linear frequency axis to allow the display of faster frequencies. p-value limits relate to adjustment for multiple comparisons.

The statistical significance of these ratios at each frequency ω was determined by deriving their probability distribution from first principles (see Supplementary Data). The significance threshold was set at α=0.05 ; however, because this test was conducted at multiple frequency ω of the frequency grid (resolution 0.33 Hz), we used false discovery rate (FDR) to correct for multiple comparisons. Moreover, we also used Bonferroni correction to account for testing at multiple channels (n = 19). The p-values threshold in Figures 1e and 2e reflects these corrections (p = 0.0026).

We then defined a group-level frequency band-specific canonical stimulus‑evoked EEG power change at five bands: slow delta (${\text{Ω}}_{1}$, 0.2–2 Hz), fast delta (${\text{Ω}}_{2}$, 2–4 Hz), theta (${\text{Ω}}_{3}$, 4–8 Hz), alpha-low beta (${\text{Ω}}_{4}$, 8–20 Hz), and high beta-gamma (${\text{Ω}}_{5}$, 20–40 Hz). For each band f, we defined the median EEG power change elicited by tactile stimulation (interpreted as canonical somatosensory network engagement) as:

$${\bar{T}}_{f}^{\left(k\right)}=\frac{\int_{{\text{Ω}}_{f}}{\bar{S}}_{S}^{\left(k\right)}\left(\omega \right)d\omega }{\int_{{\text{Ω}}_{f}}{\bar{S}}_{B}^{\left(k\right)}\left(\omega \right)d\omega }.$$ (4)

### 2.6 Analysis of spontaneous EEG somatosensory network activity effect on stimulus-evoked EEG power changes

We next investigated whether spontaneous activation of somatosensory networks influences their stimulus-driven recruitment, and whether any such effect reflects a refractory-like process with a limited temporal duration and frequency-related characteristics.

Because ongoing activity cannot be experimentally controlled, we addressed these questions by exploiting natural trial-by-trial variability in baseline activity. Specifically, we assessed (i) the degree to which somatosensory networks were active prior to each stimulus, (ii) whether such spontaneous activation modulated the subsequent stimulus-evoked response, (iii) whether this modulation persisted when spontaneous somatosensory activity occurred further from stimulus onset, and (iv) whether these effects varied across frequency bands.

#### 2.6.1 Detection of spontaneous activation of somatosensory networks preceding stimulation

To assess whether somatosensory networks were spontaneously active prior to each stimulus, we quantified the similarity between the spatial distribution of baseline EEG power and the canonical stimulus‑evoked EEG power change defined in Section 2.5. Specifically, for each subject i, trial j, and frequency band f, we compared the topographical distribution of baseline EEG power ${\hat{S}}_{B,ijf}$ with the group-level stimulus‑evoked EEG power change ${\bar{T}}_{f}$. A closer match between the two was interpreted as stronger evidence that somatosensory cortical networks were spontaneously active prior to stimulus delivery. We first define the EEG power at each frequency band f preceding each stimulus at channel k as:

$${\hat{S}}_{B,ijf}^{\left(k\right)}=\int_{{\text{Ω}}_{f}}{\hat{S}}_{B,ij}^{\left(k\right)}\left(\omega \right)d\omega .$$ (5)

Baseline-template similarity was then quantified using cosine similarity in channel space:

$$sim_{ijf}=\frac{\langle {\hat{S}}_{B,ijf}^{\left(k\right)},{\bar{T}}_{f}^{\left(k\right)}\rangle }{{\left\|{\hat{S}}_{B,ijf}^{\left(k\right)}\right\|}_{k}{\left\|{\bar{T}}_{f}^{\left(k\right)}\right\|}_{k}}$$ (6)

The frequency band-specific baseline-template similarity index $sim_{ijf}$ ranges from 0 to 1, with 0 indicating no similarity between spatial topographies and 1 indicating an exact match.

To obtain a single summary measure of baseline somatosensory networks engagement for each trial, we summed the frequency-specific baseline-template similarity indices across all frequency bands:

$$sim_{ij}=\sum_{f}sim_{ijf}$$ (7)

$sim_{ij}$ ranges from 0 to 5 as we considered five frequency bands (see Section 2.5).

#### 2.6.2 Assessing the effect of spontaneous activation of somatosensory networks on their engagement by a stimulus

We next tested whether spontaneous activation of somatosensory networks immediately preceding stimulation influenced their subsequent stimulus-driven recruitment. To this end, we examined the relationship between the baseline-template similarity index $sim_{ij}$ and the magnitude of the stimulus-evoked EEG power change ${\hat{T}}_{ij}$ . The stimulus-evoked response was quantified as the Euclidean norm in channel space of the channel-wise EEG power change ${\hat{T}}_{ij}^{\left(k\right)}$, expressed in decibels:

$${\hat{T}}_{ij}=10log_{10}{\left\|{\hat{T}}_{ij}^{\left(k\right)}\right\|}_{k},$$ (8)

Where k indexes EEG channels and

$${\hat{T}}_{ij}^{\left(k\right)}=\sum_{f}{\hat{S}}_{S,ijf}^{\left(k\right)}/\sum_{f}{\hat{S}}_{B,ijf}^{\left(k\right)}.$$ (9)

${\hat{S}}_{S,ijf}^{\left(k\right)}$ and ${\hat{S}}_{B,ijf}^{\left(k\right)}$ denote the post-stimulus and pre-stimulus EEG power, respectively, for trial j, subjects i at frequency band f and channel k as defined in (5).

The relationship between ${\hat{T}}_{ij}$ and $sim_{ij}$ was assessed with a mixed-effects linear regression model, with repeated trials j clustered within subjects i. Models were implemented using package *lme4* (Bates et al., 2015) in R v.4.4 (R Core Team, 2024). By explicitly modelling the correlation between trials within subjects (intraclass correlation, ICC), this approach provides appropriate estimates of standard errors. The model was specified as:

$${\hat{T}}_{ij}=(\beta_{0}+b_{0i})+\beta_{1}sim_{ij}+\beta_{2}PMA_{ij}+\beta_{3}sleep_{ij}+\;\varepsilon_{ij}$$ (10)

where $b_{0i}$ denotes a subject-specific random intercept (random effect) and $\varepsilon_{ij}$ the residual error term.

In (10), we have controlled for postmenstrual age (PMA ) and quiet or active sleep state (sleep ) as they are the primary source of inter-individual variance in the neonatal EEG (Leikos et al., 2020; Whitehead et al., 2016). To assess whether including $sim_{ij}$ improved model fit compared to a model containing only PMA and sleep , we used Akaike Information Criterion (AIC), which penalises model complexity by accounting for the number of estimated parameters. Lower AIC values indicate better model fit, with differences greater than 10 units considered substantial (Portet, 2020). We also compared total pseudo-R^2^ (random and fixed effects) and fixed-effects pseudo-R^2^ (fixed effects only) between models using function summ.merMod in R package *jtools* (Long, 2022) (The “pseudo-R^2^” terminology reflects that in mixed-effects models, variance includes random and fixed effects). We used R package *effects* to visualise the effect of sim on $\hat{T}$ after correcting for PMA and sleep (Fox & Weisberg, 2019). We report the effect of *PMA* on *$\hat{T}\;$ *, and *sleep* on *$\hat{T}\;$ *, after likewise correcting for the other two variables, in Supplementary Data.

Although both the baseline-template similarity measure $sim_{ij}$ and the stimulus‑evoked EEG power change ${\hat{T}}_{ij}$ involve baseline data, they quantify different aspects of the EEG signal by construction. Baseline similarity reflects the spatial configuration of pre‑stimulus EEG power across channels and is computed using cosine similarity, making it invariant to absolute power magnitude. In contrast, the stimulus‑evoked response reflects the magnitude of EEG power change between post‑ and pre‑stimulus epochs and does not incorporate any measure of spatial similarity. Thus, these measures capture distinct degrees of freedom—direction versus length of the EEG power vector in channel space—and there is no deterministic relationship between them. In addition, baseline similarity is evaluated relative to a fixed, group‑level somatosensory response pattern, which does not incorporate trial‑specific baseline or evoked activity and serves solely as an external descriptor of somatosensory network engagement.

#### 2.6.3 Assessing whether the effect of spontaneous activation of somatosensory networks on their engagement is temporally constrained

We next tested whether the influence of spontaneous somatosensory network activation on stimulus-driven recruitment was temporally constrained. To this end, we repeated the analysis described in Section 2.6.2 using baseline EEG segments progressively further removed in time from stimulus onset. If the observed relationship between baseline similarity and stimulus-evoked power change reflects a refractory-like phenomenon, its influence should diminish with increasing temporal separation between baseline activity and stimulation.

Specifically, we computed the baseline similarity index using EEG power extracted from an earlier pre-stimulus interval spanning 6 to 3 s before stimulus onset, and one temporally unrelated to the stimulus, rather than the period immediately preceding stimulation (3 to 0 s before stimulus onset). We then assessed whether similarity derived from these more temporally distant baselines exhibited a comparable relationship with stimulus-evoked EEG power changes.

Baselines temporally unrelated to the stimulus were obtained by randomly shuffling baseline EEG segments 6 to 3 s before stimulus onset across trials prior to computing similarity. This procedure preserves the overall distribution of baseline spectro-spatial patterns while disrupting their trial-wise temporal correspondence with stimulus-evoked responses. This effectively simulates baseline activity further removed from the stimulus.

#### 2.6.4 Assessing whether the effect of spontaneous activation of somatosensory networks on their engagement is frequency specific

Finally, we tested whether the influence of spontaneous somatosensory network activity on stimulus-driven recruitment—and its temporal constraint—was frequency specific. To this end, we repeated the analyses described above separately for each frequency band. Stimulus-evoked EEG power change was quantified for each band f as:

$${\hat{T}}_{ijf}=10log_{10}{\left\|{\hat{T}}_{ijf}^{\left(k\right)}\right\|}_{k},$$ (11)

where

$${\hat{T}}_{ijf}^{\left(k\right)}={\hat{S}}_{S,ijf}^{\left(k\right)}/{\hat{S}}_{B,ijf}^{\left(k\right)}.$$ (12)

and k  indexes EEG channels.

The relationship between band-specific stimulus-evoked power change ${\hat{T}}_{ijf}$ and similarity $sim_{ijf}$ was assessed using the same model in (10). This results in five separate regressions: for slow delta (0.2–2 Hz), fast delta (2–4 Hz), theta (4–8 Hz), alpha-low beta (8–20 Hz), and high beta-gamma (20–40 Hz). As the dependent variables of these regressions are related, we used Bonferroni correction to adjust the p-values threshold (0.05/5, i.e., 0.01). The temporal specificity analysis was then repeated within each frequency band by (i) computing frequency-specific similarity from the earlier pre-stimulus interval (6 to 3 s before stimulus onset), and (ii) shuffling the corresponding baseline EEG segments across trials prior to similarity computation, exactly as described above.

## 3 Results

### 3.1 Somatosensory-evoked slow neuronal activity is widespread across the scalp, while fast activity engages local somatotopically specific areas

We first defined the spectro-spatial characteristics of somatosensory-evoked activity in 35 preterm infants of median 32 weeks postmenstrual age (Table 1). Tactile stimulation of hands and feet evoked wideband power increases across all scalp EEG channels (Figs. 1 and 2) with two distinct spectral peaks within the slow delta band (1 Hz) and alpha-low beta band (13 Hz) (Figs. 1c and 2c; see Supplementary Data and Supplementary Fig. S4 for quantification).

The topographic distribution of these power changes was frequency dependent. Significant power changes (compared to baseline) at slower frequencies were widespread across the scalp (Figs. 1e and 2e). Changes at faster frequencies were focused over the somatotopically appropriate scalp regions—that is, contra-lateral following hand stimulation (e.g., note the power change at high beta-gamma in the hemisphere contralateral to stimulus compared to that in the ipsilateral hemisphere) and midline central channels following feet stimulation (Fig. 1d and e and Fig. 2d and e; see Supplementary Data and Supplementary Fig. S5 for further quantification).

### 3.2 Spontaneous activity of somatosensory networks dampens EEG power changes evoked by tactile stimulation

**Fig. 3.**
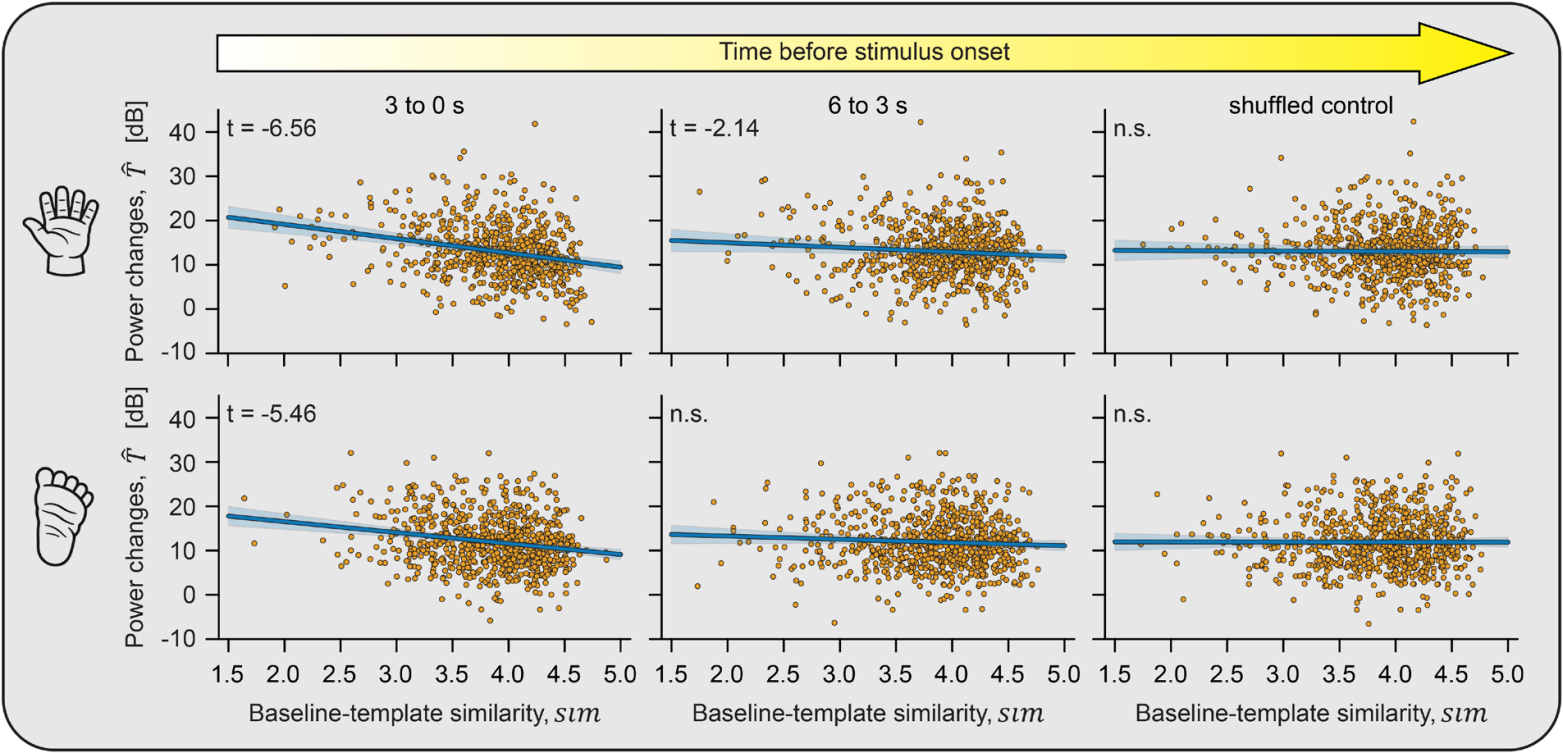
Temporally constrained relationship between baseline-template similarity and stimulus-evoked power changes. Partial regression plots showing evoked power changes ($\hat{T}$ ) following hand and foot stimulation as a function of baseline-template similarity (sim ) computed over three pre-stimulus periods: 3 to 0 s and 6 to 3 s before stimulus onset, and a shuffled 6 to 3 s before stimulus onset control (simulating baseline activity further removed from the stimulus). Residuals are derived from the full regression model including postmenstrual age (PMA), sleep state, and similarity; corresponding partial effects for PMA and sleep are shown in Supplementary Figure S6. The solid blue line represents the fitted partial regression, with shaded areas indicating 95% confidence intervals for the mean. Each point represents a single trial.

We then sought to determine whether the spontaneous activity of somatosensory cortical networks influenced their recruitment by external stimulation. Since we had no control over the ongoing activity at the time of stimulation, we examined whether these networks happened to be spontaneously active prior to each stimulus. To assess this, we evaluated how baseline activity with spectro-spatial characteristics similar to those evoked by somatosensory stimulation (i.e., a group-defined somatosensory-evoked template) influenced the power changes elicited by the stimulus. Specifically, we quantified the similarity between the baseline EEG power distribution and the somatosensory-evoked template for subject i, trial j (baseline-template similarity, $sim_{ij}$ ), and examined how this similarity related to the EEG power change evoked by the corresponding stimulus (${\hat{T}}_{ij}$ ) (Fig. 3). Baseline-template similarity could range between 0 and 5, where 0 is no similarity between topographies and 5 is an exact match (see Section 2.6.1). A close match between the spectro-spatial distribution of baseline power to the somatosensory-evoked template would imply that the somatosensory cortical networks were spontaneously active before the stimulus.

We found that higher baseline-template similarity was associated with smaller stimulus-evoked power changes. Power changes were significantly affected by the spontaneous activity during the 3 to 0 s preceding stimulus onset as demonstrated by the model including sim performing better in explaining the variance in $\hat{T}$ than a model containing *PMA* and *sleep* only (hands: Δ AIC=39.8 ; feet: Δ AIC=26.8 ; see Supplementary Data and Supplementary Fig. S6 for the statistics relating to *PMA* and *sleep*)*.* Power changes were more similar within infant than between infants (hands: ICC = 0.14; feet: ICC = 0.04). Including sim relative to the model containing *PMA* and *sleep* only explained an additional 0.05 pseudo-R^2^ fixed effects for the hands and 0.03 pseudo-R^2^ fixed effects for the feet (specifically, including sim increased total pseudo-R^2^ from 0.27 to 0.32 and fixed effects pseudo-R^2^ from 0.15 to 0.20 for hands, and total pseudo-R^2^ from 0.11 to 0.13 and fixed effects pseudo-R^2^ from 0.06 to 0.09 for feet). Stimulus-evoked power changes decreased by 3.2 dB (hands, t = -6.56) and 2.5 dB (feet, t = -5.46) per 1.0 degree increase in similarity (i.e., 20% of total similarity range, see Supplementary Fig. S7 for illustrative examples). This meant that, for the hands, stimulus-evoked power changes at sim = 1.9 (min), 2.6, 3.3, 4, and 4.7 (max) were 19.4, 17.2, 14.9, 12.7, and 10.4 dB, with *PMA* and *sleep* held at their mean. For the feet, stimulus-evoked power changes at sim = 1.6 (min), 2.4, 3.3, 4.1, and 4.9 (max) were 17.5, 15.5, 13.3, 11.3, and 9.4 dB. These relationships could not be explained by a positive correlation between baseline-template similarity and baseline power (see Supplementary Data and Supplementary Fig. S8).

We next tested whether the influence of spontaneous somatosensory network activity on stimulus-driven recruitment was temporally constrained. To this end, we repeated the baseline-template similarity analysis using EEG segments progressively further removed from stimulus onset. For hands, as described above, stimulus-evoked power decreased by 3.2 dB per 1.0 degree increase in similarity to baseline immediately preceding stimulation (3 to 0 s before stimulus onset). This effect was attenuated when similarity was computed from the earlier baseline 6 to 3 s before stimulus onset (stimulus-evoked power decreased by 1.0 dB; t = −2.14) and was abolished when baseline segments from the same interval were randomly shuffled across trials, thereby approximating a baseline that is temporally unlinked—and effectively further removed—from the stimulus (-0.1 dB; n.s.; Fig. 3).

A similar pattern was observed for foot stimulation. As described above, stimulus-evoked power decreased by 2.5 dB per 1.0 degree increase in similarity to the baseline 3 to 0 s before stimulus onset, but this relationship was not statistically significant when similarity was computed from 6 to 3 s before stimulus onset (-0.7 dB but n.s.: t = −1.71) and clearly absent when baseline segments from the same interval were randomly shuffled across trials (0.0 dB; n.s.; Fig. 3).

### 3.3 Spontaneous activity of somatosensory networks dampens stimulus‑evoked EEG power changes in a frequency‑dependent manner

We then examined whether the relationship between baseline-template similarity and stimulus-evoked EEG power changes was frequency dependent. Overall, the baseline-template similarity index was lowest in the slow delta band (median: 0.74 for hands, 0.71 for feet) and tended to be higher in faster frequencies, particularly fast delta and theta (medians: 0.85–0.86 for hands and 0.84–0.84 for feet) (band-specific similarity ranges from 0 to 1 as described in Section 2.6.1).

Across frequency bands, greater baseline-template similarity to the baseline immediately preceding stimulation (3 to 0 s before stimulus onset) was consistently associated with smaller stimulus-evoked power changes. This effect was evident for all bands for hand stimulation, except for high beta–gamma (t values ranging from −7.26 to −4.36, with high beta-gamma: t = −0.43), and across all bands for foot stimulation (t values ranging from −6.07 to −3.58).

The strength of this relationship varied by frequency, with the largest effect observed in fast delta, followed by slow delta, theta, alpha–low beta, and high beta–gamma. For these bands, power decreased by 2.7 (fast delta), 2.4 (slow delta), 1.9 (theta), 1.9 dB (alpha-low beta), and n.s. for high beta-gamma for the hands, and by 2.6, 2.0, 1.8, 1.5, and 0.8 dB, respectively, for the feet, per 0.2 degree increase in similarity (i.e., 20% of band-specific similarity range).

**Fig. 4.**
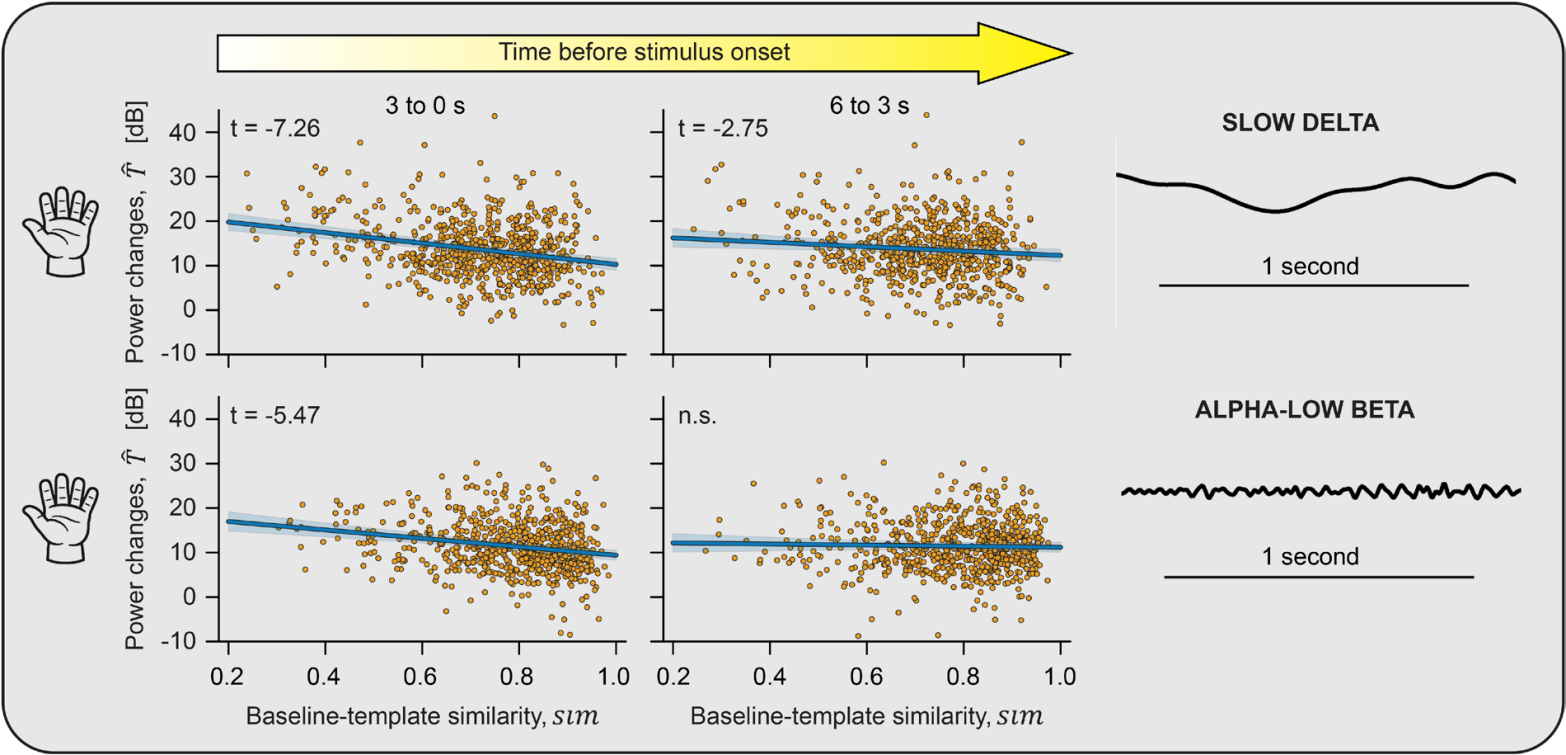
The relationship between baseline-template similarity and stimulus-evoked power changes is frequency-dependent. Partial regression plots showing evoked power changes ($\hat{T}$ ) following hand stimulation as a function of baseline-template similarity (sim in the slow delta band, computed over two pre-stimulus periods (3 to 0 s and 6 to 3 s before stimulus onset)). The same relationship is shown for the alpha-low beta band for comparison. Residuals are derived from the full model, including postmenstrual age (PMA), sleep state, and similarity. The solid blue line represents the fitted partial regression, with shaded areas indicating 95% confidence intervals for the mean. Each point represents a single trial.

This relationship was temporally constrained. Only baseline-template similarity in the slow delta band remained predictive of stimulus-evoked power changes following hand stimulation when computed from the period 6 to 3 s before stimulus onset, although with a smaller effect size. Compared to stimulus-evoked power decreasing by 2.4 dB per 0.2 degree increase in similarity to baseline immediately preceding stimulation (3 to 0 s before stimulus onset) (t = -7.26), this effect was attenuated when similarity was computed from the earlier interval (6 to 3 s before stimulus onset) with power decreasing by 1.0 dB per 0.2 degree increase in similarity (t = −2.75, p = 0.0062; Fig. 4). No significant relationships were observed for any other frequency band, nor for the shuffled control condition, following either hand or foot stimulation.

## 4 Discussion

We demonstrated that tactile stimulation in preterm human neonates elicits a wideband increase in EEG power, but that the level of this increase is dependent on the spectro-spatial characteristics of the activity preceding the stimulus: the closer the spectro-spatial baseline cortical activity is to that evoked by the somatosensory stimulus, the weaker is the change elicited. This effect has limited temporal duration at higher frequencies, but is more prolonged at the slowest frequencies. These results suggest that when somatosensory networks are spontaneously active, they become temporarily less responsive to stimulation – a form of refractoriness—preventing immediate reactivation in preterm neonates. This result supports the possibility that the discontinuous pattern of cortical activity at this age (burst-quiescence) may be due to network refractoriness that recovers during inter-burst intervals (Vyazovskiy et al., 2009).

### 4.1 Tactile stimulation evokes focal and extended cortical activity changes in preterm infants

External tactile (Leikos et al., 2020; Milh et al., 2007; Vanhatalo et al., 2009; Whitehead et al., 2018), noxious (Fabrizi et al., 2011; Green et al., 2018), visual (Colonnese et al., 2010), and auditory (Chipaux et al., 2013; Kaminska et al., 2018) stimuli elicit rudimentary cortical responses from as early as 28 weeks gestation in preterm human infants. These responses are generally composed of fast ripples overriding a larger slow wave focussed around the scalp locations overlaying the primary sensory areas relevant to stimulus modality (i.e., central – tactile; occipital – visual). Here, we show that these responses (to tactile stimuli) have rich spectro-spatial characteristics, where oscillations at different frequencies have different voltage fields. The tactile response had a wideband power spectrum but included two clear peaks within the alpha-low beta and slow delta range and a dip in the theta range. Such coupled oscillatory signatures are a hallmark of activity-dependent processes at time scales relevant for synaptic plasticity (Buzsáki et al., 2013). Changes above 20 Hz (in the high beta-gamma range) were localized to central contralateral and midline areas following hand and foot stimulation respectively, while those at the slow end of the spectrum were more widespread.

These results could imply analogous mechanisms to those observed in rodent pups, in which somatosensory stimulation elicits distinct patterns of oscillations which differentially synchronise neonatal cortical networks (Yang et al., 2009). Early Gamma Oscillations (EGO) involve focal networks limited to a single cortical column or barrel (McVea et al., 2012; Minlebaev et al., 2011; Yang et al., 2013), while long oscillations extend beyond those boundaries (Yang et al., 2009). EGO are driven by thalamic oscillators and are thought to support the development of thalamocortical projections and drive the organization of vertical topographic functional units (Minlebaev et al., 2011), while long oscillations may serve as a tangential signal binding these units together within the somatomotor area (An et al., 2014; Quairiaux et al., 2011; Yang et al., 2009). As thalamo-cortical connections’ formation precedes that of cortico-cortical connections in the human brain (Volpe, 2009), a role for slow oscillations in synchronizing cortico-cortical ensembles may underlie why sensory-evoked delta activity declines later in preterm infants than fast ripples (Chipaux et al., 2013; Whitehead et al., 2018). Our results suggest that sensory evoked gamma activity may represent the focal activation of columnar functional sensory units, while slow activity may represent horizontal coordination between them within the contralateral somatomotor area (Allievi et al., 2016).

### 4.2 Spontaneous engagement of somatosensory network suppresses their externally-driven excitability

Cortical bursts in preterm infants can be evoked by sensory stimulation, but can also occur spontaneously in the absence of external stimuli (Whitehead et al., 2016) (Supplementary Video 2). These spontaneous activity patterns occur with varying spatial configurations (Arichi et al., 2017) which overlap with those evoked by external stimulation (Supplementary Fig. S1). Here, we show that the response to a somatosensory input is suppressed if the background cortical activity preceding stimulus onset resembles that evoked by the stimulus itself.

In neonatal animal models, spontaneous activity starts from central pattern generators in the sensory periphery, spinal cord, thalamus, or subplate (Martini et al., 2021). Independently of their source, when these patterns propagate to the cortex, they ultimately involve the same regions which are engaged by external stimuli, and are confined within the domain related to one sensory modality (e.g., somatosensory: (Mizuno et al., 2018; Nakazawa et al., 2020); visual: (Kenet et al., 2003; O’Hashi et al., 2018; Ruthazer & Stryker, 1996; Smith et al., 2018)). Concordant with this, in preterm human infants, resting-state fMRI activity involves the same areas engaged by sensory stimulation, suggesting that these same networks can also be coherently active in the absence of any external stimulus (Allievi et al., 2016; Doria et al., 2010). As specific patterns of cortical activation correspond to specific patterns of voltage field distribution over the scalp (Arichi et al., 2017), background EEG with spectro-spatial characteristics similar to that evoked by somatosensory stimulation is likely to represent the spontaneous unsolicited activation of developing somatosensory networks.

Whether spontaneous or evoked, early activity patterns are supported by a recurrent excitatory loop which comprises subplate neurons: a transient neuronal population in the immature brain, projecting to and receiving glutamatergic input from layers 4–6 of the cortical plate (Tolner et al., 2012; Viswanathan et al., 2012). Such a recurrent excitatory circuit will encounter activity-dependent synaptic depression, possibly because of transmitter depletion and ligand-induced changes in postsynaptic currents (Fedirchuk et al., 1999; Tabak et al., 2000). This depletion will transiently reduce network excitability, which then slowly recovers allowing for the next neuronal event, whether spontaneous or evoked, to occur. Our results, therefore, suggest that the spontaneous activation of somatosensory networks in preterm human neonates has a major dampening impact on the processing of later ascending sensory input. Importantly, however, this interpretation does not imply that these effects arise solely from subplate–cortical circuitry. As outlined above, spontaneous patterned activity in the developing nervous system can originate from multiple levels, including the sensory periphery, spinal cord, thalamus, subplate, and cortex (Martini et al., 2021), indicating that burst–quiescence dynamics represent system-level properties of developing sensory networks. Consequently, the intermittent and discontinuous nature of early activity is likely to reflect interactions across this distributed system rather than a single local mechanism. In this context, the suppression effects reported here are best interpreted as one manifestation of transient, distributed changes in excitability that can arise at multiple levels of the sensory pathway and collectively shape the processing of incoming sensory input.

Notably, the effect of baseline-template similarity was strongest and most temporally sustained at slower frequencies. Activity in the delta band not only showed the largest suppression of stimulus-evoked responses but also retained a significant influence when baseline similarity was computed from an earlier time window. In contrast, higher-frequency activity exhibited weaker and more transient effects. Within this framework, the more prolonged suppression observed at slower frequencies may reflect longer recovery times of distributed network states, while the rapid decay of effects at higher frequencies is consistent with shorter timescales of local (potentially columnar) circuit dynamics. Together, these findings suggest that the temporal extent of refractory-like modulation is not uniform across the cortex but depends on the spatial scale of the underlying networks, as indexed by their dominant frequency of activation.

This work has some limitations. EEG channel density was low relative to what is customary in older age groups, although it exceeds several existing studies in preterm infants (e.g. n = 8 channels in (Milh et al., 2007) and (Leikos et al., 2020)). In addition, the sensory stimulus was relatively uncontrolled, because given manually. This means that there will be variance in the position of exact contact with the limb, and the force applied (the coefficient of variation using the tendon hammer employed in this study is approximately 0.27 (Whitehead et al., 2019)). However, alternative protocols that can be fully standardized—such as electrical and thermal stimulation—are unethical for non-clinically required neonatal research protocols as they can extend into the nociceptive range (Bentley et al., 2003; Davis et al., 1995). Finally, a further limitation is that the similarity metric does not explicitly capture the temporal relationship between spontaneous activity and stimulus onset. Consequently, elevated similarity values may, in part, reflect stimuli delivered during ongoing spontaneous activity rather than activity that has fully resolved prior to stimulation. However, this distinction does not alter the interpretation of our findings, as both scenarios reflect a cortical baseline that is already engaged at the time of stimulation. As such, the observed modulation of stimulus-evoked responses can still be understood as a consequence of baseline-dependent, refractory-like dynamics shaping sensory processing.

In conclusion, we offer evidence that sensory-evoked activity in preterm human neonates represents the coordinated activation of local (columnar) and extended (tangential) cortical aggregates and that the occurrence of spontaneous cortical events in the same regions depresses their excitability preventing their immediate re-engagement. These results support the possibility that the discontinuous pattern of cortical activity in preterms (burst-quiescence) may be due to transient network refractoriness that recovers during inter-burst intervals.

## Supplementary Material

Supplementary Material

Supplementary Video 1

Supplementary Video 2

## Data Availability

The data and its analysis code are open-access: https://doi.org/10.17605/OSF.IO/MW35Y (Whitehead & Fabrizi, 2026).
