# Supplementary material for: Spontaneous activation of cortical somatosensory networks depresses their excitability in preterm human neonates
Source: Imaging Neurosci (Camb). 2026 Aug 6;4:IMAG.a.1324. doi: 10.1162/IMAG.a.1324 (PMC13449929; doi:10.1162/IMAG.a.1324)
Supplement: Supplementary file 1 [file IMAG.a.1324_supp.pdf]

Spontaneous activation of cortical somatosensory networks depresses their excitability in preterm human neonates

Kimberley Whitehead[1], Maria Pureza Laudiano-Dray[2], Judith Meek[3], Sofia Olhede[4], Lorenzo Fabrizi[2]

[1] Research Division of Digital Health and Applied Technology Assessment (DHATA), King's College London, SE1 8WA, London, United Kingdom
[2] Department of Neuroscience, Physiology and Pharmacology, University College London, WC1E 6BT, London, United Kingdom
[3] Elizabeth Garrett Anderson Obstetric Wing, University College London Hospitals, London, WC1E 6DB, United Kingdom
[4] Department of Mathematics, Ecole Polytechnique Federale de Lausanne, 1015 Lausanne, Switzerland

Corresponding Authors: l.fabrizi@ucl.ac.uk; kimberley.whitehead@kcl.ac.uk

Other Authors: m.laudiano-dray@ucl.ac.uk; judith.meek@nhs.net; sofia.olhede@epfl.ch

Statistical test to assess significance of stimulus-evoked EEG PSD changes

To assess the statistical significance of changes in EEG power spectral density (PSD) $\bar{T}^{(k)}(\omega) = \bar{S}_S^{(k)}(\omega)/\bar{S}_B^{(k)}(\omega)$ between baseline and post-stimulus periods, we derived the sampling distribution of the median PSD ratio from first principles. Because PSD estimates were obtained on a trial-by-trial basis using a periodogram approach, and because medians rather than means were used to aggregate across trials, the resulting test statistic does not admit a standard parametric form. We therefore capitalised on the known probabilistic properties of individual periodogram estimates to construct an analytic description of the distribution of median PSD estimates and of their ratio between post and pre-stimulus periods.

To test the significance of median PSD changes following stimulation, we first need to characterise the statistical distribution of single-trial PSD estimates. Under the null hypothesis, the observed EEG signal within each analysis window is modelled as a realisation of a stationary Gaussian process. It follows that, for a given channel k, frequency ω, and trial l, the periodogram-based PSD estimate $\hat{S}_{B,l}^{(k)}(\omega)$ (or equivalently $\hat{S}_{S,l}^{(k)}(\omega)$) can be expressed as a random variable $U(\omega)$ whose distribution is known analytically once normalised by the underlying true power spectral density. Specifically,

$$U = \frac{\hat{S}_{B,l}^{(k)}(\omega)}{\mathrm{S}_B^{(k)}(\omega)} \sim \frac{1}{2}\chi_2^2 \qquad \text{(S1)}$$

where $\mathrm{S}_B^{(k)}(\omega)$ denotes the true baseline PSD at channel k and frequency ω. The same distribution applies to the post-stimulus estimate $\hat{S}_{S,l}^{(k)}(\omega)$. Note that the true PSDs $\mathrm{S}_B^{(k)}(\omega)$ and $\mathrm{S}_S^{(k)}(\omega)$ are introduced here only as a conceptual normalisation, they cancel out in the ratio-based test statistic under the null hypothesis of $\mathrm{S}_B^{(k)}(\omega) = \mathrm{S}_S^{(k)}(\omega)$.

Since a chi-squared random variable with two degrees of freedom scaled by one half is equivalent to a unit-mean exponential distribution, the probability density function (pdf) of U is:

$$f_U(u) = e^{-u} \quad , \quad u > 0 \qquad \text{(S2)}$$

This forms the basis for deriving the sampling distribution of median PSD estimates across trials and, subsequently, of their ratio between baseline and post-stimulus conditions.

The median $\bar{U}$ of a sample of N independent realisations of U with pdf in Eq. (S2), i.e. the order statistic $U_{(N/2)}$ assuming N is even, has the following pdf:

$$\begin{aligned} f_{\bar{U}}(u) &= \frac{N!}{\left(\frac{N}{2}-1\right)!\left(\frac{N}{2}\right)!}(1-e^{-u})^{\frac{N}{2}-1}(e^{-u})^{\frac{N}{2}}e^{-u} = \\ &= \frac{N!}{\left(\frac{N}{2}-1\right)!\left(\frac{N}{2}\right)!}(1-e^{-u})^{\frac{N}{2}-1}e^{-\left(\frac{N}{2}+1\right)u} \end{aligned} \quad , \quad u > 0 \qquad \text{(S3)}$$

Similarly, if N is odd, the median corresponds to the $(N+1)/2$-th order statistic and its pdf is:

$$f_{\bar{U}}(u) = \frac{N!}{\left(\frac{N+1}{2}-1\right)!\left(N-\frac{N+1}{2}\right)!}(1-e^{-u})^{\frac{N+1}{2}-1} e^{-\left(\left(N-\frac{N+1}{2}\right)+1\right)u} \quad , \quad u > 0 \tag{S4}$$

Eqs. (S3) and (S4) for N even or odd are similar only with different parameter choices.

We finally consider the ratio of interest:

$$Y_1 = \frac{\bar{S}_S^{(k)}(\omega)}{\bar{S}_B^{(k)}(\omega)}, \tag{S5}$$

which is the ratio between two independent random variables of the form of Eq. (S3), derived from baseline and post-stimulus segments, which are treated as independent under the null hypothesis. The cumulative distribution function (CDF) of (S5) is computed using a variable substitution and numerical integration. We set:

$$Y_1 = \frac{\bar{U}_S}{\bar{U}_B} \quad Y_2 = \bar{U}_B, \tag{S6}$$

where $\bar{U}_B$ and $\bar{U}_S$ denote the medians of the normalised periodogram values for the baseline and post-stimulus segments, respectively. We now need to make a two-dimensional change of variables and integrate out over one variable at a time. We do this by inverting the relationship to obtain that:

$$\bar{U}_B = Y_2 \quad \bar{U}_S = Y_1 Y_2, \tag{S7}$$

From first principles, the joint pdf of Y_1 and Y_2 is:

$$f_{Y_1Y_2}(y_1, y_2) = |J| \cdot f_{\bar{U}}(y_1y_2) \cdot f_{\bar{U}}(y_2) \tag{S8}$$

We then calculate the Jacobian of this transformation, which is equal to Y_2, and obtain that

$$f_{Y_1Y_2}(y_1, y_2) = y_2 \cdot \left[\frac{N!}{\left(\frac{N}{2}-1\right)!\left(\frac{N}{2}\right)!}\right]^2 (1-e^{-y_1y_2})^{\frac{N}{2}-1} e^{-\left(\frac{N}{2}+1\right)y_1y_2} (1-e^{-y_2})^{\frac{N}{2}-1} e^{-\left(\frac{N}{2}+1\right)y_2} \tag{S9}$$

In practice, the evaluation of the joint density in Eq. (S9) involves factorial terms of the form $N!/(N/2-1)!\,(N/2)!$, which rapidly exceed machine precision for large N. To ensure numerical stability we approximate the factorial terms using Stirling's approximation. Specifically, the joint pdf is evaluated in log-space by writing it as the sum of a combinatorial term and a function of (y_1, y_2), and exponentiating only after simplification. This approximation preserves the functional form of the distribution while avoiding numerical overflow.

The marginal distribution of Y_1, which is our ratio of interest, is then calculated by the numerical integration:

$$f_{Y_1}(y_1) = \int_0^\infty f_{Y_1 Y_2}(y_1, y_2) dy_2 \tag{S10}$$

Finally, in order to test for the significance of $\bar{T}^{(k)}(\omega)$ we need to conduct a second numerical integration along y_1, yielding to the CDF of $Y_1 = \frac{\bar{U}_S}{\bar{U}_B}$:

$$F_{Y_1}(y_1) = \int_0^{y_1} f_{Y_1}(t) dt \tag{S11}$$

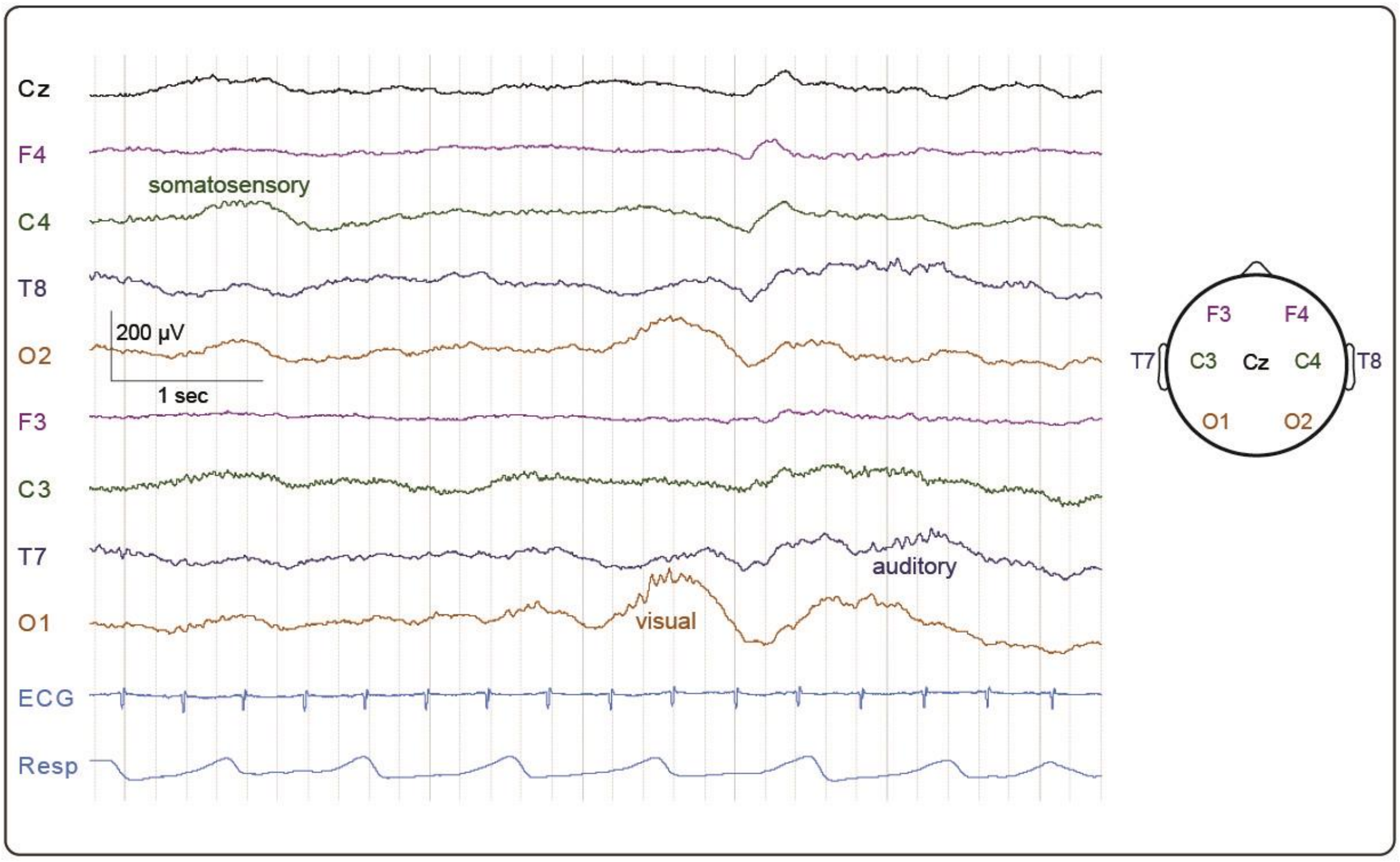

Figure S1. Spontaneous activity bursts occur with varying topographical configurations which sometimes resemble those evoked by external stimulation. Here, topographies resemble those evoked by somatosensory ((Leikos et al., 2020; Milh et al., 2007; Whitehead et al., 2018) and Fig. 3 in (Whitehead et al., 2016)), visual (Colonnese et al., 2010), and auditory (Chipaux et al., 2013; Kaminska et al., 2018) stimuli. EEG data are band-pass filtered from 0.1-70Hz for display purposes, Fz reference. This infant is 34 weeks postmenstrual age.

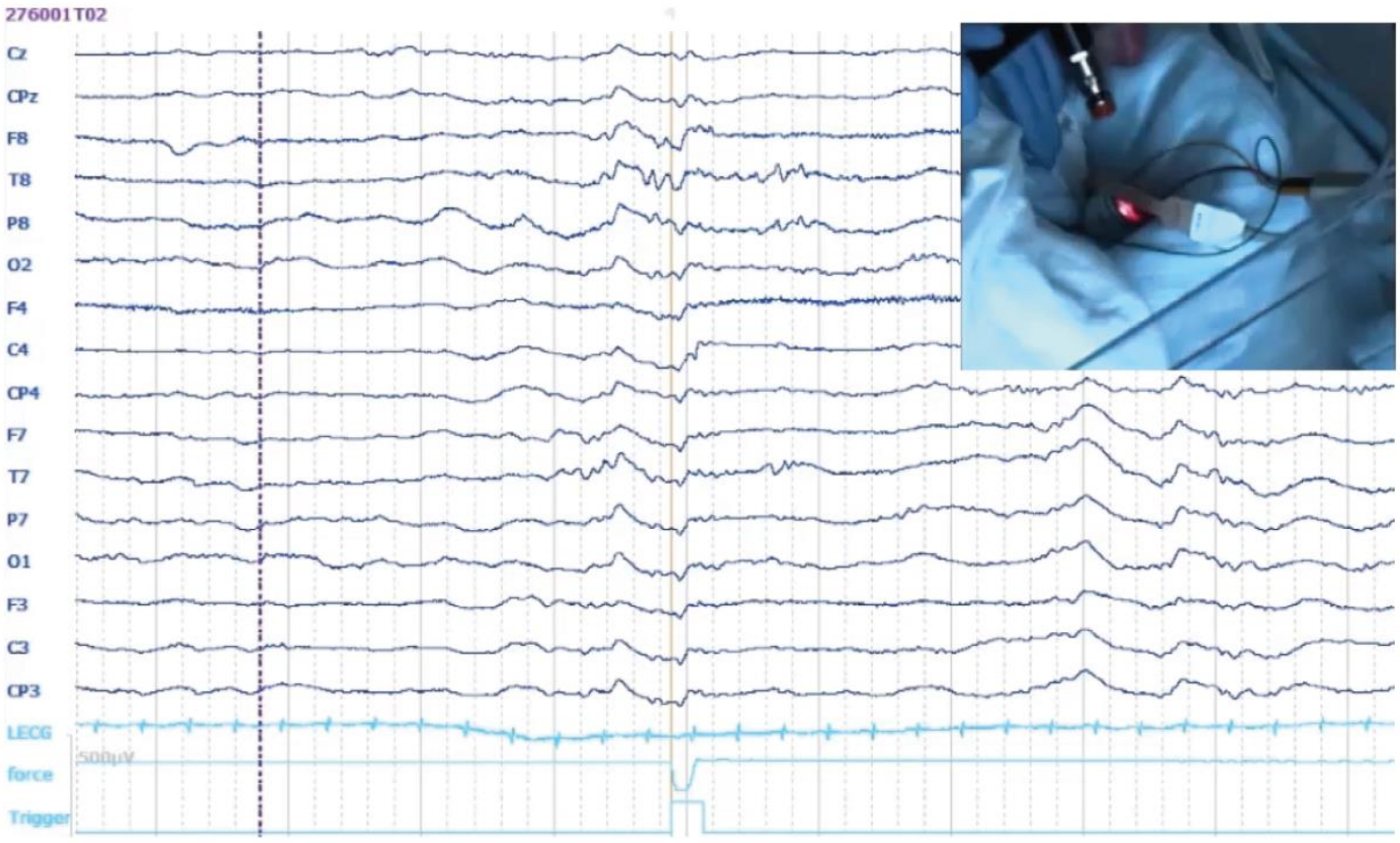

Figure S2. Stimulus timings were recorded using an impedance head with a built-in calibrated force transducer that determined the time of stimulation (orange vertical line; Trigger). In case a stimulation event was not detected by the hardware, it was identified by thresholding the force signal recorded alongside the EEG. This is an adapted still from Supplementary Video 1 in (Whitehead et al., 2020). The purple vertical line indicates the point of the EEG that the synchronised video, visible top right, is currently time-locked to. The red light in the video image is from a pulse oximetry sensor attached to the infant's foot. LECG = Left ECG.

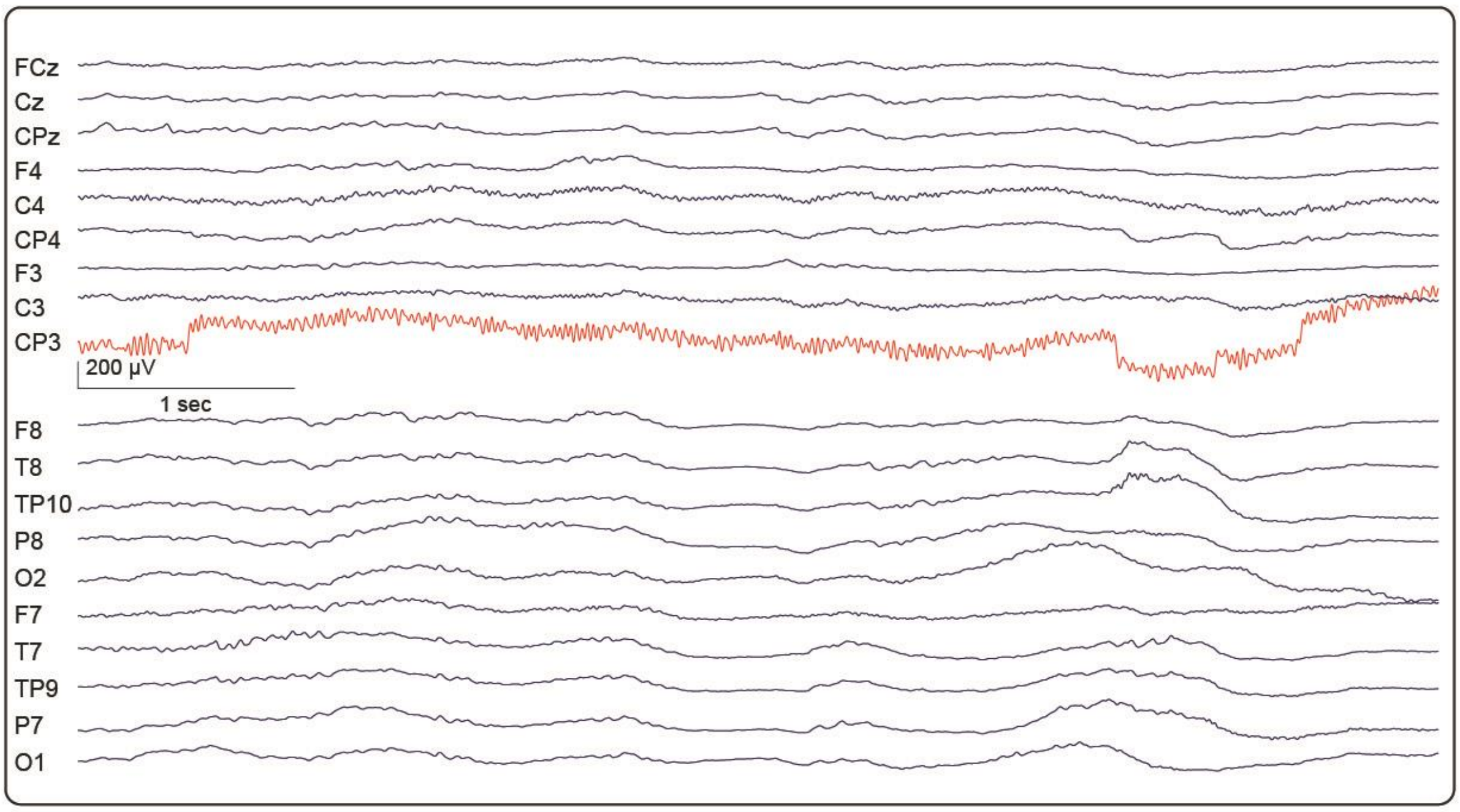

Figure S3. Illustrative data showing a bad channel (red; CP3) that was deleted and interpolated. Note how the bad channel has high-frequency noise, and 'square-like' deflections, not present on other channels (i.e. not a physiological voltage field). Data are band-pass filtered from 0.2-40Hz for display purposes, to match the frequency range analysed, Fz reference. This infant is 35 weeks postmenstrual age.

Table S1. The order in which the limbs were stimulated varied across subjects

	**Overall**, N = 101 (100%)	**T01**, N = 35 (35%)	**T02**, N = 33 (33%)	**T03**, N = 21 (21%)	**T04**, N = 12 (12%)
**Stimulation site**					
Left hand	24 (24%)	7 (20%)	9 (27%)	4 (19%)	4 (33%)
Left heel	24 (24%)	10 (29%)	6 (18%)	6 (29%)	2 (17%)
Right hand	24 (24%)	6 (17%)	8 (24%)	7 (33%)	3 (25%)
Right heel	29 (29%)	12 (34%)	10 (30%)	4 (19%)	3 (25%)

Distribution of stimulation sites by stimulation train order. Values indicate the number (percentage) of subjects receiving stimulation at each site (left hand, left heel, right hand, right heel) as the first (T01), second (T02), third (T03), or fourth (T04) stimulation train. A stimulation train refers to all taps delivered to a given limb. There was no evidence that stimulation sites were systematically assigned to a particular point in the stimulation order (Pearson's Chi-squared test with simulated p-value (2000 replicates): $\chi^2 = 4.88$, $p = 0.8486$).

Supplementary to Results 3.1: Quantification of two distinct spectral peaks in stimulus-evoked PSD increases following tactile stimulation of the hands and feet.

In Results 3.1, we describe two distinct spectral peaks in stimulus-evoked PSD increases following tactile stimulation of the hands and feet. To quantify this, we first mean averaged PSD change (Fig. 1c and 2c) across channels and identified peaks as local spectral maxima larger than the 0.7 quantile of the PSD change distribution and separated by at least 3 Hz, using R package *pracma* (Borchers, 2025) (hands: 1.0 Hz at 4.1 dB, 12.7 Hz at 3.3 dB; feet: 1.0 Hz at 2.5 dB, 13.3 Hz at 2.2 dB). A two-Gaussian model with components centred on these peaks provided a substantially better fit to the PSDs than a single-Gaussian model (hands: $\Delta AIC = 62.0$; feet $\Delta AIC = 17.0$) (Portet, 2020) (Fig. S4).

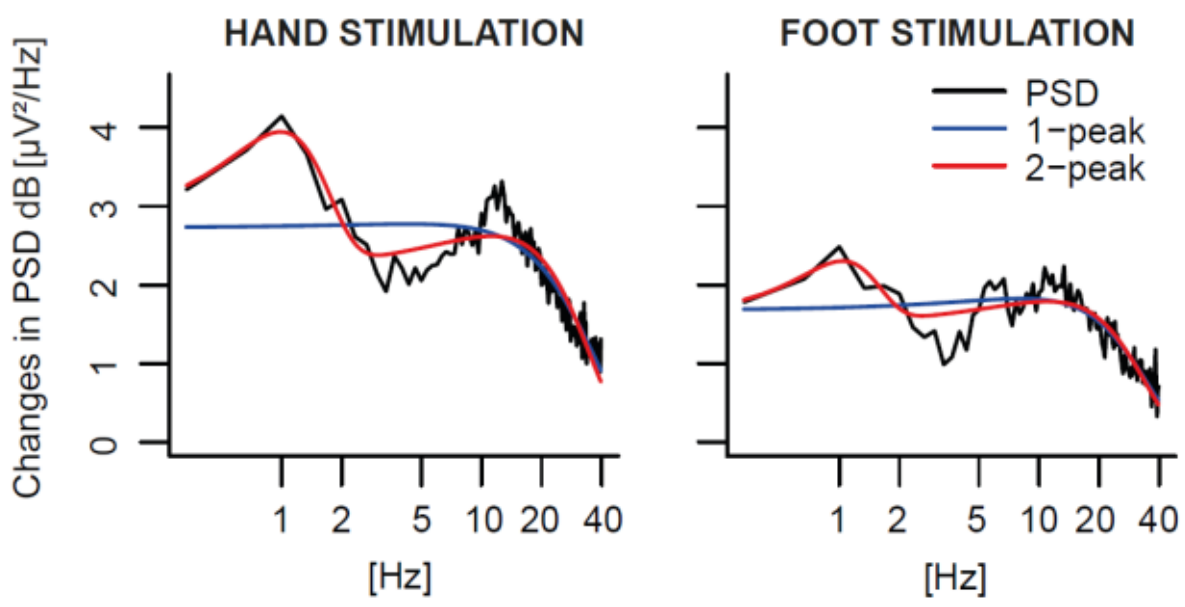

Figure S4. A two-Gaussian model provided a better fit to the stimulus-evoked PSD increases, consistent with a bimodal structure. Black traces show stimulus-evoked changes in PSD following hand and foot stimulation, mean averaged across channels. Blue lines indicate single-Gaussian model fits, while red lines indicate two-Gaussian model fits.

Supplementary to Results 3.1: Stimulus-evoked EEG power changes at slower frequencies were widespread across the scalp, while those at faster frequencies were somatotopically focussed

In Results 3.1, we observe that significant power changes (compared to baseline) at slower frequencies were widespread across the scalp, while changes at faster frequencies were focussed over the somatotopically appropriate scalp regions (Fig. 1c and d; Fig. 2c and d). To further quantify this, for stimulus-evoked power changes across the 19 EEG channels for each frequency band, we calculated both the coefficient of variation (standard deviation/mean) and the finite-sample-corrected bimodality coefficient. The latter ranges from 0 (i.e., unimodal: changes evenly distributed around a common mean, indicating a widespread effect) to 1 (i.e., bimodal: changes forming two distinct clusters), with 0.55 the conventional threshold to indicate deviation from unimodality (Pfister et al., 2013).

Stimulus-evoked power changes were more evenly distributed across channels at lower frequencies and less evenly distributed across channels at higher frequencies (Fig. S5 upper panel). This was reflected in an increasing coefficient of variation across channels from the slow delta to high beta-gamma band (hands: 0.24, 0.18, 0.20, 0.31, 0.42; feet: 0.25, 0.29, 0.30, 0.39, 0.54). In line with this, the bimodality coefficient was highest for high-beta gamma and approached the threshold to indicate deviation from unimodality (hands: 0.55, feet: 0.52). The coefficient of variation increased by 0.011 (95% CI: 0.003 0.020, $p = 0.0240$) (hands) and 0.015 (95% CI: 0.012 0.018, $p = 0.0006$) (feet) with every extra 1.0 Hz (Fig. S5 lower left panel). The bimodality coefficient increased by 0.019 (95% CI: 0.012 0.027, $p = 0.0039$) (hands) and 0.017 (95% CI: 0.003 0.030, $p = 0.0280$) (feet) with every extra 1.0 Hz (Fig. S5 lower right panel). In the regressions above, frequency band was indexed by its lower edge (e.g. 0.2 Hz for slow delta) but we confirmed that the results were also statistically significant if indexed by its average or upper edge (e.g. 1.1 Hz or 2 Hz for slow delta).

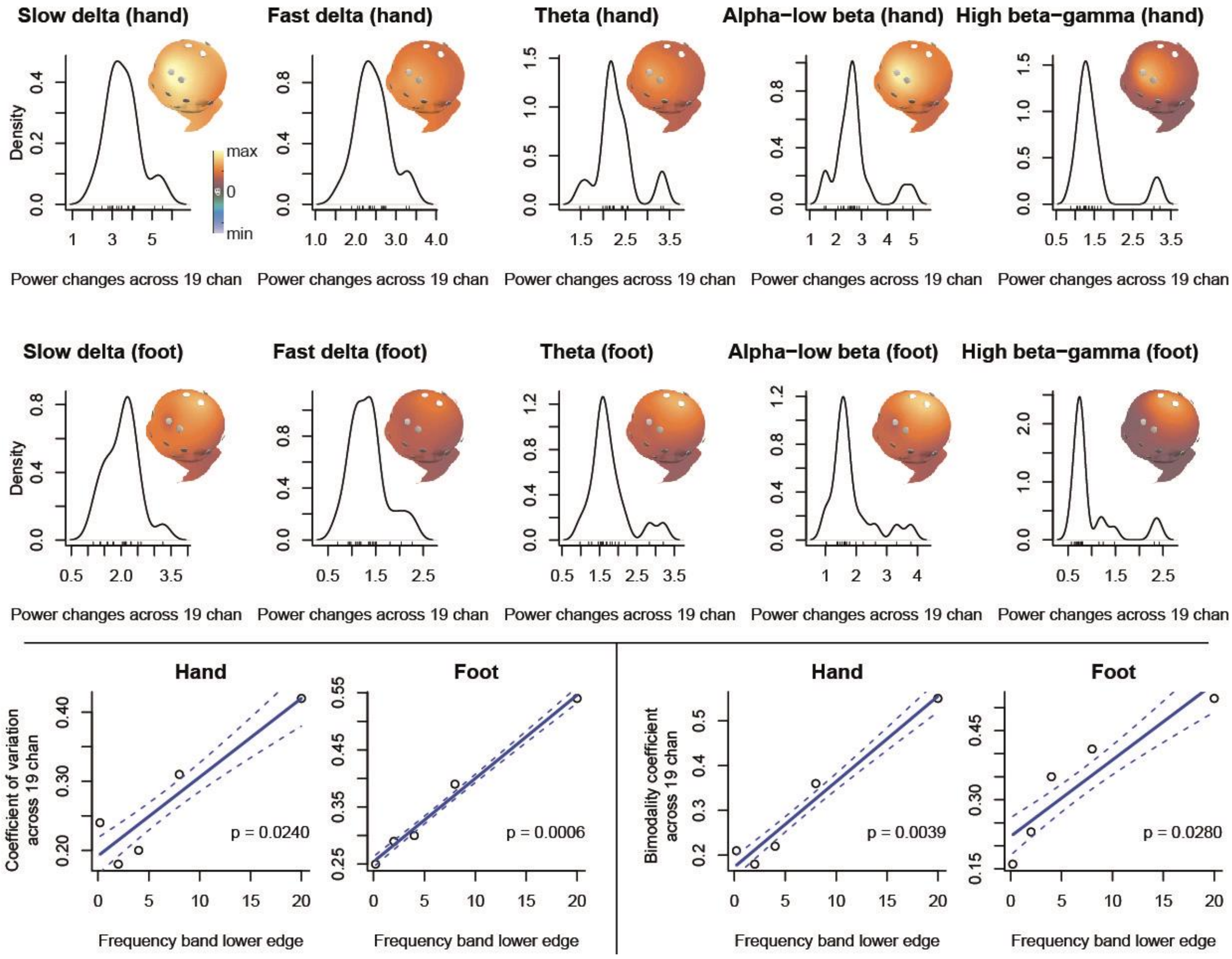

Figure S5. EEG power changes at slower frequencies were widespread across the scalp, while changes at faster frequencies were focussed. Upper panel: Probability density of power changes (dB) across 19 channels, derived using the base R density() function with a Gaussian kernel and automatic bandwidth selection (bw = 0.1898 dB) (R Core Team, 2024). The 3D maps of the scalp distribution of power changes elicited by the stimulation in the slow delta, fast delta, theta, alpha-low beta and high beta-gamma band are taken from Figure 1 and 2. Lower panel: Coefficient of variation and bimodality coefficient for power changes across the 19 EEG channels. The solid blue line is the regression fit (dashed blue lines represent the 95% confidence bounds for the mean). chan = channels.

Supplementary to Results 3.2: Higher PMA, and active relative to quiet sleep, alongside higher sim_{ij}, were associated with lower EEG power changes to tactile stimulation

For the hands, stimulus-evoked power changes decreased by 0.7 dB for every extra 1 week of PMA (t = -3.48) and 3.2 dB in active sleep relative to quiet sleep (t = -4.40) (Fig. S6), and 3.2 dB for every extra 1.0 degree of similarity 3 to 0 s before stimulus onset (t = -6.56) as described in the main text (Fig. 3).

For the feet, stimulus-evoked power changes decreased by 0.2 dB for every extra 1 week of PMA (n.s.: t = -1.63 but retained in model for consistency with the hand model) and 2.7 dB in active sleep relative to quiet sleep (t = -5.67) (Fig. S6), and 2.5 dB for every extra 1.0 degree of similarity 3 to 0 s before stimulus onset (t = -5.46) as described in the main text (Fig. 3).

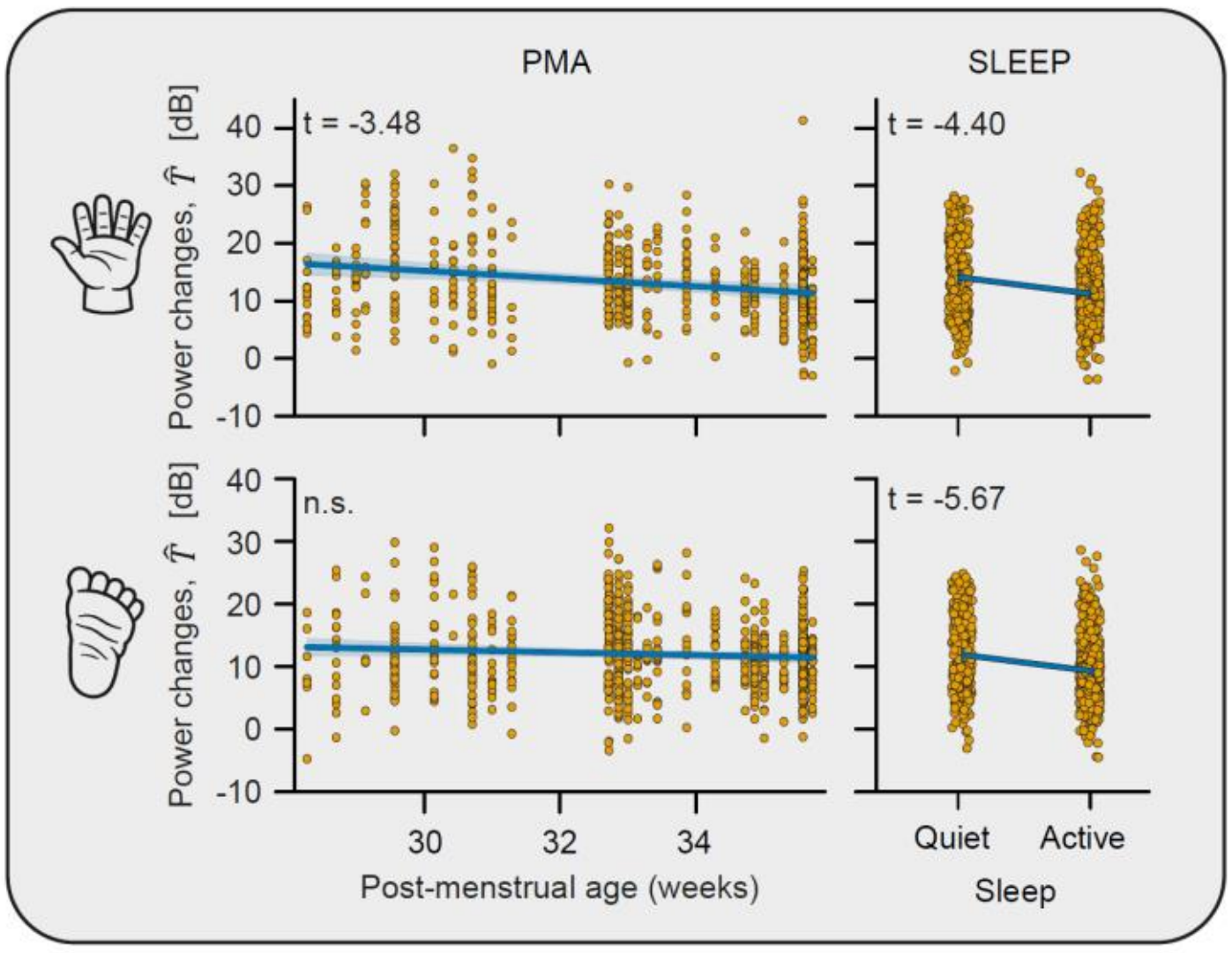

Figure S6. PMA and sleep state influenced EEG power changes evoked by tactile stimulation. Partial regression plots showing evoked power changes ($\hat{T}$) following hand and foot stimulation as a function of postmenstrual age (PMA) and sleep state. Residuals are derived from the full regression model including PMA, sleep state, and baseline-template similarity at 3 to 0 s before stimulus onset. The solid blue line represents the fitted partial regression (pale blue shading (continuous variable) or error bars (categorical variable) represent the 95% confidence bounds for the mean). Each point represents a single trial.

During 15/101 stimulation trains, the infant changed sleep state (9 quiet to active sleep; 6 active to quiet sleep). Therefore, we also conducted a sensitivity analysis in which we built the same model, but sleep state was indexed at the *end* of the stimulation train (vs. the beginning of stimulation train as above). The effect of sleep state was almost identical (power changes decreased by 3.4 dB (hands) and 2.4 dB (feet) in active relative to quiet sleep, although model fit was poorer (hands: $\Delta AIC = +3.7$; feet: $\Delta AIC = +14.0$).

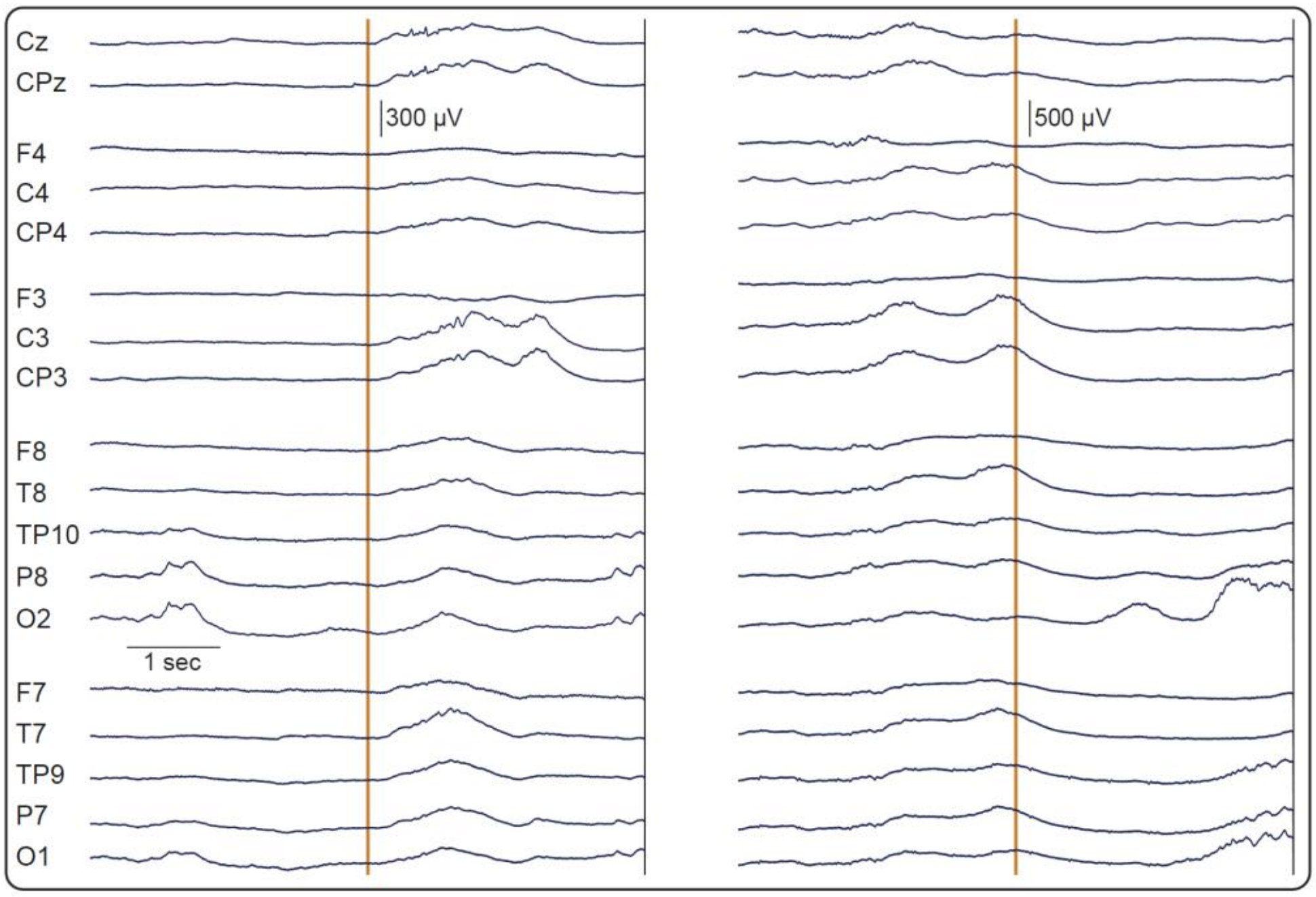

Fig. S7. Illustrative examples of the relationship between baseline-template similarity and stimulus-evoked EEG power changes. Left panel: Lower baseline-template similarity (1 centile in this example) predicts higher EEG power changes (96 centile) elicited by tapping the right foot. Right panel: Higher baseline-template similarity (98 centile in this example) predicts lower EEG power changes (41 centile) elicited by tapping the left foot. All centiles were calculated separately for hands and feet. Data are un-filtered (DC offset has been removed), Fz reference. The time of stimulation is denoted by the orange vertical line.

Supplementary to Result 3.2: baseline-template similarity and baseline power were not positively correlated

The observed negative association between baseline-template similarity (sim_{ij}) and stimulus-evoked EEG power changes ($\hat{T}_{ij}$) could, in principle, arise if baseline-template similarity were positively correlated with baseline EEG power, which appears in the denominator of the calculation of $\hat{T}_{ij}$.
However, baseline EEG power was negatively associated with sim_{ij} decreasing by 2.0 dB (hands, t = -4.82) and 1.3 dB (feet, t = -3.12) per 1.0 degree increase in similarity (i.e. 20% of total similarity range) (Fig. S8). Consistent with this, including baseline EEG power as an additional covariate in the mixed-effects model (Eq. 10) strengthened, rather than attenuated, the relationship between baseline-template similarity and stimulus-evoked power changes. Under this model, stimulus-evoked EEG power changes decreased by 5.0 dB (hands t = -13.83; 3.2 dB without baseline EEG power in the model) and 3.4 dB (feet t = -9.64; 2.5 dB without baseline EEG power in the model) per 1.0 degree increase in similarity. The negative association between baseline–template similarity and baseline EEG power was observed across all frequency bands except for fast delta. Baseline EEG power decreased by 1.0 to 2.7 dB (hands, t values ranging from -3.86 to -17.51, with fast delta: t = -2.45) and 1.2 to 2.2 dB (feet, t values ranging from -4.23 to -13.36, with fast delta: t = -1.46) per 0.2 degree increase in similarity (corresponding to 20% of the band-specific similarity range). Accordingly, including baseline EEG power as an additional covariate in the mixed-effects model strengthened, rather than attenuated, the relationship between baseline–template similarity and stimulus-evoked power changes across the spectrum. Stimulus-evoked EEG power changes decreased by 3.8 (slow delta), 3.8 (fast delta), 3.4 (theta), 3.0 (alpha-beta) and 2.1 dB (high beta-gamma) for the hands, and by 3.1, 3.0, 2.7, 2.7 and 2.2 dB, respectively, for the feet, per 0.2 degree increase in similarity when baseline EEG power was included in the model.
The negative association between baseline-template similarity and baseline EEG power may partly reflect the predominance of high-power posterior temporo-occipital bursts during the preterm period (Arichi et al., 2017), whose scalp topography poorly matches that of somatosensory-evoked activity (Fig. S7 left panel). Consequently, periods of high baseline power would be often associated with lower similarity to the somatosensory template.

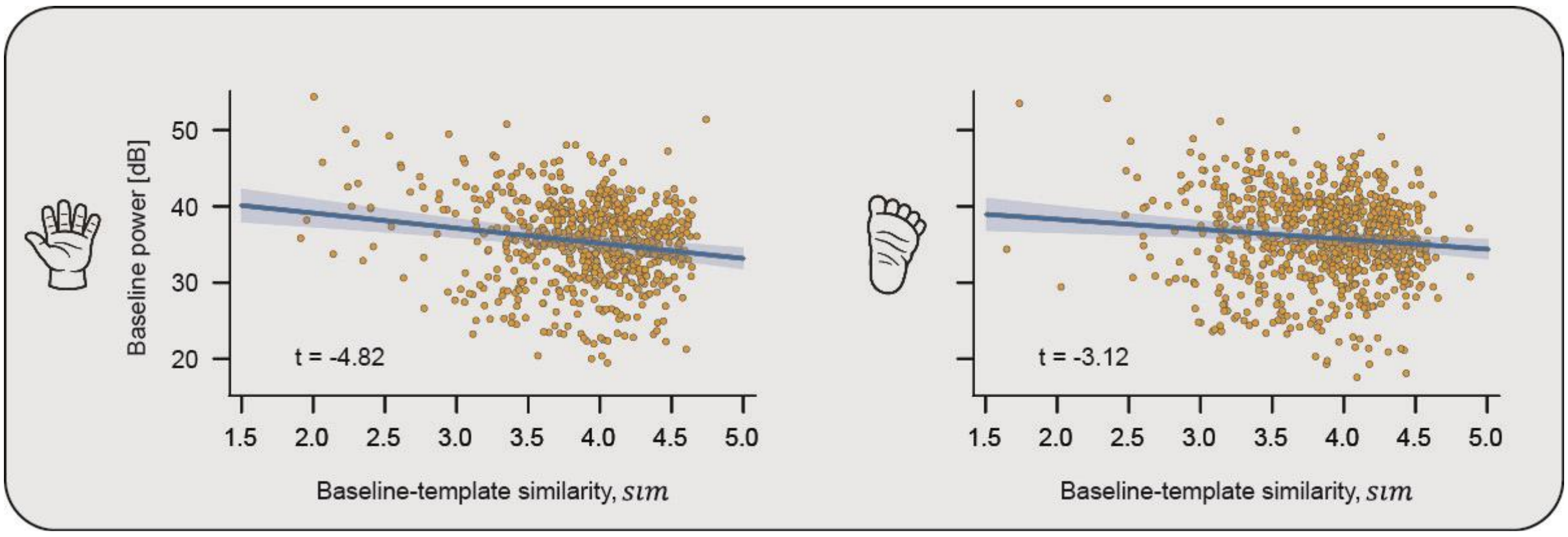

Figure S8. Baseline EEG power was not positively but negatively correlated with baseline-template similarity. Regression plot showing baseline EEG power as a function of baseline-template similarity, at 3 to 0 s before stimulus onset. The solid blue line represents the fitted regression (pale blue shading represents the 95% confidence bounds for the mean). Each point represents a single trial.

Supplementary Video

Supplementary Video 1: Two examples of tactile stimulation of the left hand in a 35 weeks postmenstrual age infant. To maintain the infant's comfort, they remain wrapped in their bedding, with only a small amount of skin uncovered to deliver the tap.

Supplementary Video 2: Example of a spontaneous cortical burst in a 31 weeks postmenstrual age infant.

Bibliography

Arichi, T., Whitehead, K., Barone, G., Pressler, R., Padormo, F., Edwards, A. D., & Fabrizi, L. (2017). Localization of spontaneous bursting neuronal activity in the preterm human brain with simultaneous EEG-fMRI. *eLife*, *6*, e27814. https://doi.org/10.7554/eLife.27814

Borchers, H. (2025). *pracma: Practical Numerical Math Functions* (Version 2.4.6) [Computer software]. https://CRAN.R-project.org/package=pracma

Chipaux, M., Colonnese, M. T., Mauguen, A., Fellous, L., Mokhtari, M., Lezcano, O., Milh, M., Dulac, O., Chiron, C., Khazipov, R., & Kaminska, A. (2013). Auditory Stimuli Mimicking Ambient Sounds Drive Temporal "Delta-Brushes" in Premature Infants. *PLoS ONE*, *8*(11), e79028. https://doi.org/10.1371/journal.pone.0079028

Colonnese, M. T., Kaminska, A., Minlebaev, M., Milh, M., Bloem, B., Lescure, S., Moriette, G., Chiron, C., Ben-Ari, Y., & Khazipov, R. (2010). A Conserved Switch in Sensory Processing Prepares Developing Neocortex for Vision. *Neuron*, *67*(3), 480–498. https://doi.org/10.1016/j.neuron.2010.07.015

Kaminska, A., Delattre, V., Laschet, J., Dubois, J., Labidurie, M., Duval, A., Manresa, A., Magny, J.-F., Hovhannisyan, S., Mokhtari, M., Ouss, L., Boissel, A., Hertz-Pannier, L., Sintsov, M., Minlebaev, M., Khazipov, R., & Chiron, C. (2018). Cortical Auditory-Evoked Responses in Preterm Neonates: Revisited by Spectral and Temporal Analyses. *Cerebral Cortex*, *28*(10), 3429–3444. https://doi.org/10.1093/cercor/bhx206

Leikos, S., Tokariev, A., Koolen, N., Nevalainen, P., & Vanhatalo, S. (2020). Cortical responses to tactile stimuli in preterm infants. *European Journal of Neuroscience*, *51*(4), 1059–1073. https://doi.org/10.1111/ejn.14613

Milh, M., Kaminska, A., Huon, C., Lapillonne, A., Ben-Ari, Y., & Khazipov, R. (2007). Rapid Cortical Oscillations and Early Motor Activity in Premature Human Neonate. *Cerebral Cortex*, *17*(7), 1582–1594. https://doi.org/10.1093/cercor/bhl069

Pfister, R., Schwarz, K. A., Janczyk, M., Dale, R., & Freeman, J. (2013). Good things peak in pairs: A note on the bimodality coefficient. *Frontiers in Psychology*, *4*. https://doi.org/10.3389/fpsyg.2013.00700

Portet, S. (2020). A primer on model selection using the Akaike Information Criterion. *Infectious Disease Modelling*, *5*, 111–128. https://doi.org/10.1016/j.idm.2019.12.010

R Core Team. (2024). *R: A language and environment for statistical computing* [Computer software]. R Foundation for Statistical Computing. https://www.R-project.org/

Whitehead, K., Jones, L., Laudiano-Dray, M. P., Meek, J., & Fabrizi, L. (2020). Altered cortical processing of somatosensory input in pre-term infants who had high-grade germinal matrix-intraventricular haemorrhage. *NeuroImage: Clinical*, *25*, 102095. https://doi.org/https://doi.org/10.1016/j.nicl.2019.102095

Whitehead, K., Meek, J., & Fabrizi, L. (2018). Developmental trajectory of movement-related cortical oscillations during active sleep in a cross-sectional cohort of pre-term and full-term human infants. *Scientific Reports*, *8*(1), 17516. https://doi.org/10.1038/s41598-018-35850-1

Whitehead, K., Pressler, R., & Fabrizi, L. (2016). Characteristics and clinical significance of delta brushes in the EEG of premature infants. *Clinical Neurophysiology Practice*, *2*, 12–18. https://doi.org/10.1016/j.cnp.2016.11.002
